# Pediatric suicide attempts lagged during the COVID-19 pandemic: a European multicenter study

**DOI:** 10.1186/s13034-024-00784-2

**Published:** 2024-08-07

**Authors:** Ana Moscoso, Anthony Cousien, Giulia Serra, Annette Erlangsen, Mar Vila, Ljubica Paradžik, Sandra Pires, Francisco Villar, Marija Bogadi, Pedro Caldeira da Silva, Stefano Vicari, Mette Falkenberg Krantz, Richard Delorme, & the ACE-COVID study group, Sarah do Amaral, Elisa Andracchio, Massimo Apicell, Ivana Bilić, Fabrizia Chieppa, Nuno Araújo Duarte, Iolanda Faustino, Madalena Ferro Rodrigues, Clotilde Guidetti, Carsten Hjorthøj, Maria Elena Iannoni, Ana Kordić, Federica Lombardini, Gino Maglio, Marianna Moro, Catarina M Nascimento, Merete Nordentoft, Maria de Oliveira Tareco, Elise Pennanec’h, David Antonio Silva, Monia Trasolini, Vincent Trebossen

**Affiliations:** 1 Child and Adolescent Psychiatry Department, Robert Debré Hospital, APHP & Université Paris Cité Paris, 48, Boulevard Sérurier, 75935 Paris Cedex 19 Paris, France; 2 Université Paris Cité and Université Sorbonne Paris Nord, Inserm, IAME, F‑75018, Paris, France; 3 Child and Adolescent Neuropsychiatry Unit, Bambino Gesù Children’s Hospital, IRCCS, Rome, Italy; 4 Danish Research Institute for Suicide Prevention, Mental Health Centre Copenhagen, Copenhagen, Denmark; 5 Department of Mental Health, Johns Hopkins Bloomberg School of Public Health, Baltimore, MD USA; 6 National Centre for Epidemiology and Population Health, Centre for Mental Health Research, The Australian National University, Canberra, Australia; 7 Child and Adolescent Mental Health Service, Hospital Sant Joan de Déu Barcelona, Barcelona, Spain; 8 Psychiatric Hospital for Children and Youth, Zagreb, Croatia; 9 Child and Adolescent Psychiatry Department, Hospital D. Estefânia, CHLC, Lisbon, Portugal; 10 Department of Life Sciences and Public Health, Catholic University, Rome, Italy; 11 Copenhagen Research Center for Mental Health– CORE, Mental Health Center Copenhagen, Copenhagen University Hospital, Copenhagen, Denmark

**Keywords:** Suicide attempts, Children and adolescents, COVID-19 duration and severity, Oxford COVID-19 government response tracker (OxCGRT)

## Abstract

**Background:**

Elevated rates of suicidal behavior were reported during the COVID-19 pandemic. However, information is scarce on patients’ profiles during this period. Studies evoke the potential adverse effects of the mandatory lockdown, but they remain relatively speculative.

**Methods:**

We monitored fluctuations in suicide attempts (SA) in six European countries. We gathered data, retrospectively for under 18-year-old SA episodes (1 January 2018 to 31 December 2021), through records of psychiatric emergency services. We collected clinical profiles individually. We extracted environmental indicators by month, as provided by Oxford COVID-19 Government Response Tracker (OxCGRT). We used the Pruned Exact Linear Time (PELT) method to identify breakpoints in SA episodes reported for each country, and logistic regressions to estimate changes in patients’ characteristics after the breakpoints. Finally, we used a univariate and multivariate negative binomial model to assess the link between SA and OxCGRT indicators, accounting for the delay (lag) between the interventions and their impact on SA.

**Results:**

The study comprised 2,833 children and adolescents (mean age = 15.1 years (SD 1.6); M: F sex-ratio = 1:5.4). A significant increase in SA was found either 6 or 10 months after the beginning of the pandemic, varying by country. Patients were more likely to be girls (aOR = 1.77 [1.34; 2.34]) and used SA methods “other than self-poisoning” (aOR = 1.34 [1.05; 1.7]). In the multivariate model, an association was found between SA and the contact tracing indicator with an 11 months delay, and the number of COVID-19 deaths with a 3-months delay.

**Conclusions:**

Findings confirmed a delayed increase in SA during the COVID-19 pandemic in children and adolescents as well as changes in patients’ profiles. The duration and severity of the pandemic emerged as the strongest predictor in the rise of SA. If faced with a similar pandemic in the future, the gap between the onset of pandemic and the increase in suicide attempts presents an opportunity for prevention.

**Supplementary Information:**

The online version contains supplementary material available at 10.1186/s13034-024-00784-2.

## Background

Increased suicidality in children and adolescents during the COVID-19 pandemic has been described, specifically among the most vulnerable. Even though the experience of lockdown was not the same for all [1], there is a compelling body of evidence highlighting increased suicidality in children and adolescents during the pandemic [2]. Early studies, published a few weeks after the imposition of containment measures, pointed towards either a decrease, or inconclusive fluctuations in suicidality [3, 4]. Later and more robust studies progressively revealed early inflections that were followed by a significant increase in the subsequent months [5–7]. The direct and indirect effects of COVID-19 infection are the source of interest as a potential precipitant for suicide attempts (SA). Social, psychological, and economic consequences that resulted from the pandemic can be accounted as potential stressful life events, even in children, and appeared as negative life events precipitating the process of suicidal ideation and behavior [8]. However, correlations between COVID-19 specific political measures to avoid the spread of the virus (e.g., relative isolation due to school closure) and suicidal spectrum remain merely speculative [9]. Previously, this was limited by the paucity of objective data to allow for comparisons between countries, which launched distinct strategies to combat the pandemic. To fill this gap, researchers from the University of Oxford developed the Oxford COVID-19 Government Response Tracker (OxCGRT), a dataset of longitudinal measures of government responses related to closure and containment, health, and economy policies during the pandemic. Data provided has previously been used to understand how policies relate to human behaviors [10]. Mental health issues have also been investigated using the OxCGRT stringency index [11, 12]. Building upon our previous work [6], our first aim was to understand how SA in children and adolescents progressed in different European countries during the COVID-19 pandemic. Second, indicators concerning the patients’ clinical profiles were collected to explore for potential variations. Third, we explored the correlations between SA fluctuations and OxCGRT environmental indicators to investigate how political measures, used by countries to avoid the spread of the virus, were related to the rise of SA.

## Methods

### Study design

In a retrospective, multicenter study, the incidence of SA recorded in child psychiatric emergency departments in six European countries during the COVID-19 pandemic was compared with that of previous years.

### Setting

We included data from child and adolescent psychiatric emergency departments in six different European countries (Croatia, Denmark, France, Italy, Portugal, and Spain; please see Supplementary Table 1 for more details) that fulfilled pre-established criteria:

1. The facility is an emergency department dedicated to the care of children and adolescents (under 18-year-old) with suicidal ideation and attempts, and other psychiatric problems.
2. The professionals in charge of the assessment are trained on identifying and managing suicidal behaviors.
3. Deserving a strategic geographical area of the concerned country, i.e. capital or major city with relatively high population density, the hospital covering regional or national health needs.

Alternatively, national hospital databases obtained from centers with the above characteristics were also accepted.

The ethical approval for the study (ACE-COVID) was first obtained in July 2021 from the Ethics Committee of Robert Debré University Hospital– AP-HP (ethics committee Approval Number: 2021-577) and subsequently by local authorities in each country.

### Data collection

Data were gathered retrospectively by experienced clinicians through digital and paper datafile records from local psychiatric emergency services. Inclusion criteria were: < 18-year-old children and adolescents who attempted suicide and were admitted at the psychiatric emergency department from January 1st, 2018–December 31st, 2021. Two countries (Portugal, Denmark) extended the period of data collection (Portugal from January 1st, 2017–December 31st, 2021, and Denmark from January 1st, 2018–May 31st, 2022).

A suicide attempt (SA) was defined as a non-fatal self-directed potential injurious behavior with any intent to die because of the behavior [13]. SA episodes were defined as SAs occurring within a 7-day interval prior to admission. If multiple SAs occurred within this period, only the characteristics of the first SA were considered for the study. Suicide attempts occurring more than 7 days prior to admission were excluded, or counted as separate SA episodes if they occurred within the study period and involved an admission to an emergency room. Deaths by suicide were not included in the analysis, as identification required data beyond our chosen setting (e.g., national records on mortality causes). The incidence of SA was calculated by month during the studied period. Denmark rendered pooled data (monthly for females and quarterly for males), so we were only able to utilize female data in our analysis. For each episode of a SA, we collected the following clinical information: age, sex, SA method, family psychiatric history, and number of previous ER visits for a SA, except for Denmark where these data could not be disseminated due to existing data security regulations.

### OxCGRT indicators covariates

Data were made freely available by authors [10] online: https://github.com/OxCGRT/covid-policy-tracker. Indicators were extracted for each studied country participating in this study, by month as follows: (1) The data on confirmed COVID-19 cases and death counts were represented on a logarithmic scale. We hypothesized that minor changes in the number of deaths, such as an increase from 100 or 300, may not significantly impact the number of SA. However, a substantial increase in deaths, such as from 100 to 1000 or 10,000 might have a noticeable effect. (2) Policy indicators of containment and closure (e.g., school closing, stay-at-home requirements, restrictions on international travel) and health (e.g., facial covering, vaccination policy) were reported in ordinal scales and were assessed as such (please see S1- Supplementary materials for details).

### Statistical analysis

*First*, we calculated the fluctuations in SA incidence for each country by analyzing mobile mean trends and monthly plots for the study period. Next, we identified points where the statistical properties (mean and variance) of the time-series of SA changed. We used the PELT (Pruned Exact Linear Time) method to identify breakpoints in monthly time-series for each country [14]. This algorithm allows the identification of one change-points using the « changepoint» package in R [15].

*Second*, we identified covariates associated with the changes detected using PELT. For each country, we determined univariate, country-specific logistic regression models. We considered the response variable as the time period using breakpoints previously identified. We then estimated multivariate logistic regression models where all covariates with p-values < 0.20 in the univariate analysis were included. We considered covariates as significant in multivariate analysis using the common threshold p < 0.05. Next, by gathering all countries data, we identified the covariates associated with the SA variations detected with the PELT by using multilevel logistic regression analysis and including a marker for the country as a random effect. We performed both univariate and multivariate analysis. For the latter, we also selected the covariates when the p-values were below 0.20 in the univariate analysis.

*Third,* we assessed SA fluctuations regarding OxCGRT indicators [10] using a negative-binomial regression model, while accounting for seasonal variation. We included all OxCGRT indicators and 3 additional variables in the model: a trend to catch a possible increase in SA not related to OxCGRT indicators during the period of our analysis, the elapsed time (in months) since the beginning of the pandemic in Europe (*i.e.* March 2020) to account for the potential deleterious impact of the sustainability of the pandemic over time, and a pandemic period indicator variable equal to 1 if the current month is after March 2020, and 0 otherwise. Next, we used a negative binomial regression model to determine time lapses between a change in an OxCGRT indicator value and the effect expected on the number of SA. We estimated the optimal time value for which the statistical link between the OxCGRT indicator and the number of SA was strongest. We called this value the “optimal lag” of the indicator. We then selected the most significant indicators by using a Bayesian Information Criteria (BIC) [16]. For each indicators’ optimal lag, we performed a forward selection: we started from the empty model including only the nuisance parameters (seasonality and overdispersion) and sought which indicator was associated with the lowest BIC when added in the model. The last model (i.e. with the lowest BIC) was used as the final multivariate model. More details are given in Supplementary Material S1.

## Results

The study comprised 2,833 children and adolescents who attempted suicide during the studied period, with a mean age of 15.1 (SD = 1.6) years and a male to female ratio of 1:5.4.

### Trends in SA and changepoint detection

**Fig. 1:**
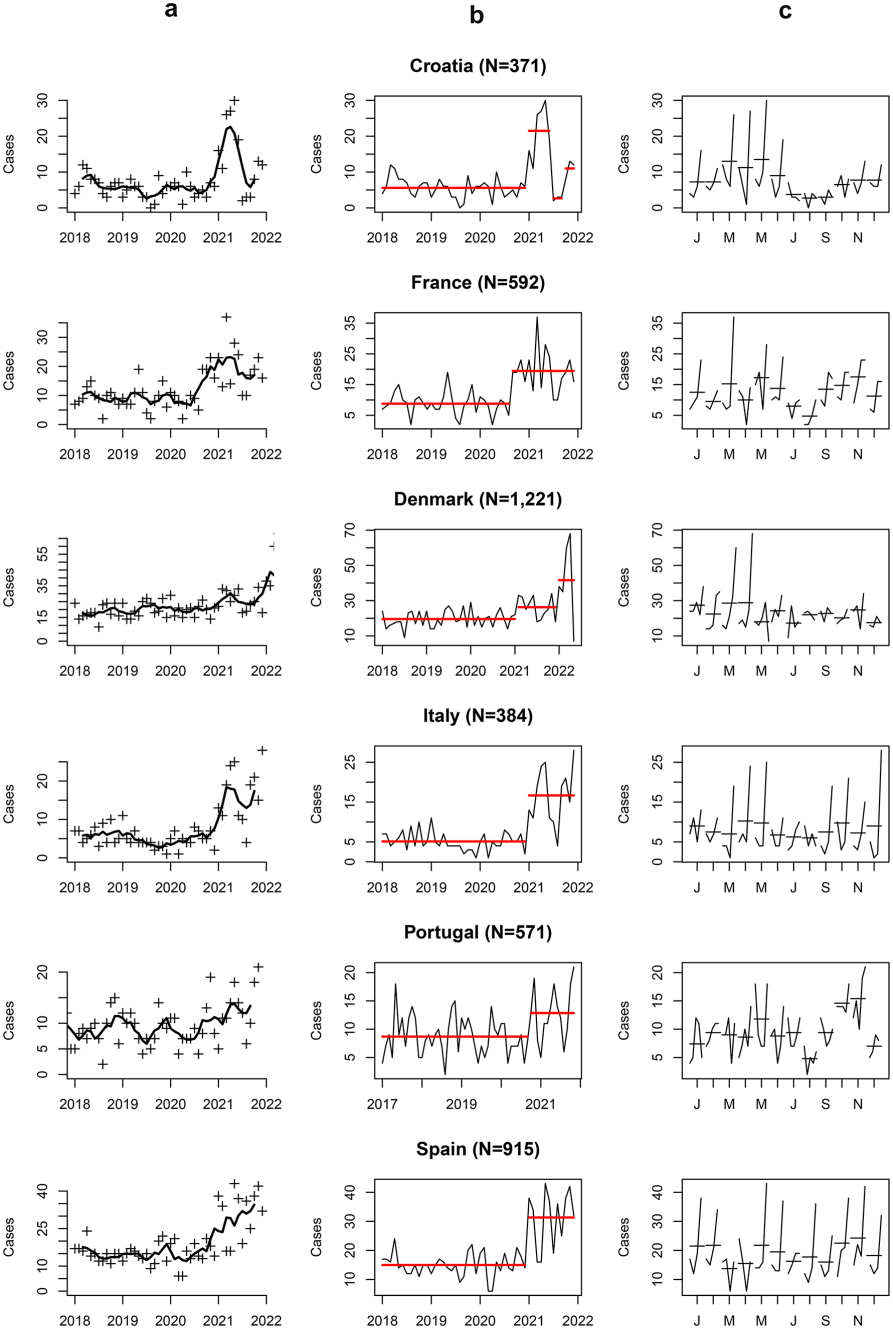
Trends in variations of suicide attempts across included countries over the studied period. Denmark fluctuations concern females only. **a** Trends as captured by the mobile mean; **b** mean rupture as measured by change point; **c** Month plot of the studied period; each subplot corresponds to a specific month (e.g., January, February, etc.). Data points within each month are shown over the study period, allowing for the comparison of the same month across different years and the identification of outliers on a month-by-month basis

Variations in SA rates over time were detected when analyzing data for each country separately (Fig. 1a). The onset of the initial lockdown occurred at approximately the same date, March 2020, for all the participating countries. A significant increase (p < 0.05) in SA was observed in all countries after lockdown, but breakpoints differed: 6 months later (September 2020) for France and Portugal, and 10 months later (January 2021), for Croatia, Denmark, Italy, and Spain (Fig. 1b; Table 1). Plots of SA by months across the studied period (Fig. 1c) provided information regarding seasonal variations. Except for spring 2020, a peak in SA incidence was observed during spring and a nadir at the end of summer.

**Table 1:** Sample description per country according to the time-period (before and after the breakpoints)

Croatia		**1st period** **01/2018–12/2020** (N = 201)	**2nd period** **01/2021–12/2021** (N = 170)	**p-value**
	Suicide attempt mean (SD)	5.6 (2.7)	14.2 (9.6)	< 2e-16
	Male—N (%)	62/201 (30.8%)	15/170 (8.8%)	1.80e-7
	Age—mean (sd)	15.7 (1.7)	15.2 (1.7)	0.015
	Previous ER visit—N (%)	96/201 (47.8%)	75/169 (44.4%)	0.52
	Family history—N (%)	73/157 (46.5%)	50/87 (57.5%)	0.11
	Method—N (%)			
	Self-poisoning	143 (71.1%)	75 (44.4%)	1.90e-7
	Other	58 (28.9%)	94 (55.6%)	
France		**1st period** **01/2018–08/2020** (N = 358)	**2nd period** **09/2020–12/2021** (N = 234)	**p-value**
	Suicide attempt mean (SD)	8.8 (3.7)	19.4 (6.9)	< 2e-16
	Male—N (%)	64/318 (20.1%)	35/220 (15.9%)	0.21
	Age—mean (sd)	14.0 (1.8)	14.4 (1.5)	8.80e-3
	Previous ER visit—N (%)	109/280 (38.9%)	99/213 (46.5%)	0.093
	Family history—N (%)	80/234 (34.2%)	86/201 (42.3%)	0.066
	Method—N (%)			
	Self-poisoning	185 (67.3%)	127 (58.0%)	0.033
	Other	90 (32.7%)	92 (52.0%)	
Italy		**1st period** **01/2018–12/2020** (N = 184)	**2nd period** **01/2021–12/2021** (N = 200)	**p-value**
	Suicide attempt mean (SD)	5.1 (2.3)	16.7 (7.2)	< 2e-16
	Male—N (%)	31/184 (16.8%)	23/199 (14.6%)	0.55
	Age—mean (sd)	15.9 (1.5)	15.6 (1.4)	0.066
	Previous ER visit—N (%)	93/183 (50.8%)	90/195 (46.2%)	0.36
	Family history—N (%)	81/113 (71.7%)	61/150 (40.7%)	0.038
	Method—N (%)			
	Self-poisoning	110 (59.8%)	139 (70.6%)	0.027
	Other	74 (40.2%)	58 (29.4%)	
Portugal		**1st period** **01/2017–09/2020** (N = 391)	**2nd period** **10/2021–11/2021** (N = 180)	**p-value**
	Suicide attempt mean (SD)	8.7 (3.4)	12.9 (4.9)	0.0354
	Male—N (%)	37/302 (12.3%)	16/150 (10.7%)	0.62
	Age—mean (sd)**	15.2 (1.4)	15.0 (1.5)	0.20
	Previous ER visit—N (%)	40/88 (45.5%)	18/30 (60.0%)	0.17
	Family history—N (%)	54/76 (71.1%)	12/30 (40.0%)	3.0e-3
	Method—N (%)			
	Self-poisoning	71 (81.6%)	17 (56.7%)	6.3e-3
	Other	16 (18.4%)	13 (43.3%)	
Spain		**1st period** **01/2018–12/2020** (N = 539)	**2nd period** **01/2021–12/2021** (N = 376)	**p-value**
	Suicide attempt mean (SD)	15.0 (4.0)	31.3 (9.8)	< 2e-16
	Male—N (%)	92/539 (17.1%)	32/373 (8.6%)	0.0002
	Age—mean (sd)	15.2 (1.6)	15.0 (1.5)	0.07
	Previous ER visit—N (%)	117/539 (21.7%)	90/376 (19.4%)	0.4
	Family history—N (%)	234/533 (43.9%)	134/367 (36.5%)	0.027
	Method—N (%)			
	Self-poisoning	453 (89.2%)	324 (91.0%)	0.038
	Other	55 (11.8%)	32 (9.0%)	
Denmark		**1st period** **01/2018–01/2021** (N = 724)	**2nd period** **02/2021–12/2021** (N = 497)	**p-value**
	Suicide attempt mean (SD)	19.6 (4.6)	31.1 (15.3)	< 2e-16

Data from Denmark were restricted to females only

*ER* emergency room

### Children’s profile before and after the peak

We aimed to assess the clinical profile of children before and after the identified SA breakpoints. For Croatia, we brought together the last three periods (i.e., from January 2020), as the decrease observed in summer 2021 seemed temporary and matched with the seasonality expected in SA time-series [17, 18]. To a lesser extent, similar patterns were observed in Italy and France but not detected by the PELT algorithm. Results, organized by covariate, are comprised of (a) country-specific logistic regression models’ findings [univariate, Odds-ratio (OR); and multivariate, adjusted-OR (aOR)] (Table 2); and (b) multilevel logistic regression models’ findings (univariate, OR; and multivariate, aOR) (Table 3).

**Table 2:** Odds-ratios with 95% confidence intervals and p-values for univariate and multivariate country-specific logistic regression models

	Univariate models		Multivariate model	
	Odds-ratio [95% confidence interval]	p-value	Adjusted odds-ratio [95% confidence interval]	p-value
	**Croatia**			
Age	0.86 [0.76; 0.97]	0.017	0.88 [0.74; 1.03]	0.11
Sex (ref = Male vs. Female)	4.61 [2.57; 8.75]	8.6e-7	6.32 [2.71; 16.91]	6.6 e-5
Previous ER visit	0.87 [0.58; 1.32]	0.52		
Family history	1.55 [0.92; 2.65]	0.1	1.45 [0.81; 2.6]	0.21
Method (ref = Self-poisoning, vs. Other)	3.09 [2.02; 4.77]	2.8e-7	3.57 [1.96; 6.66]	4.3e-5
	**France**			
Age	1.15 [1.03; 1.28]	0.013	1.2 [1.06; 1.36]	0.0055
Sex (ref = Male vs. Female)	1.33 [0.85; 2.11]	0.22	–	–
Previous ER visit	1.36 [0.95; 1.96]	0.093	0.96 [0.64; 1.44]	0.85
Family history	1.44 [0.98; 2.13]	0.066	1.36 [0.9; 2.05]	0.15
Method (ref = Self-poisoning, vs. Other)	1.49 [1.03; 2.15]	0.034	1.84 [1.21; 2.83]	0.0049
	**Italy**			
Age	0.88 [0.76; 1.01]	0.068	0.76 [0.63; 0.91]	0.004
Sex (ref = Male vs. Female)	1.19 [0.68; 2.07]	0.54	–	–
Previous ER visit	1.21 [0.81; 1.81]	0.36	–	–
Family history	0.58 [0.34; 0.97]	0.039	0.59 [0.34; 1]	0.051
Method (ref = Self-poisoning, vs. Other)	0.62 [0.4; 0.95]	0.028	0.6 [0.35; 1.01]	0.053
	**Portugal**			
Age	0.92 [0.82; 1.04]	0.19	1 [0.7; 1.43]	1
Sex (ref = Male vs. Female)	1.17 [0.64; 2.23]	0.62		
Previous ER visit	1.8 [0.78; 4.26]	0.17	1.22 [0.47; 3.14]	0.68
Family history	0.27 [0.11; 0.65]	0.0038	0.32 [0.12; 0.81]	0.017
Method (ref = Self-poisoning, vs. Other)	3.39 [1.37; 8.45]	0.008	2.62 [0.95; 7.2]	0.06
	**Spain**			
Age	0.92 [0.85; 1.01]	0.071	0.93 [0.86; 1.02]	0.13
Sex (ref = Male vs. Female)	2.19 [1.45; 3.4]	3.0e-4	2.13 [1.4; 3.32]	5.5e-4
Previous ER visit	0.87 [0.62; 1.2]	0.4		
Family history	0.73 [0.56; 0.96]	0.027	0.73 [0.55; 0.96]	0.025
Method (ref = Self-poisoning, vs. Other)	0.81 [0.51; 1.28]	0.38	–	–

Variables with p < 0.20 in the univariate analysis were included in the multivariate model

*ER* emergency room

**Table 3:** Odds-ratios with 95% confidence intervals and p-values for univariate and multivariate multilevel logistic regression models

	Univariate models		Multivariate model	
	Odds-ratio [95% confidence interval]	p-value	Adjusted odds-ratio [95% confidence interval]	p-value
Age	0.96 [0.91; 1]	0.064	0.96 [0.91; 1.02]	0.22
Sex (ref = Male vs. Female)	1.82 [1.45; 2.29]	0.00000021	1.77 [1.34; 2.34]	0.00006
Previous ER visit	1.11 [0.93; 1.32]	0.26	–	–
Family history	0.87 [0.73; 1.05]	0.15	0.92 [0.76; 1.12]	0.43
Method (ref = Self-poisoning, vs. Other)	1.34 [1.1; 1.63]	0.0041	1.34 [1.05; 1.7]	0.018

We included in the multivariate model the variables with p < 0.20 in the univariate analysis *ER* emergency room

#### Age

Country specific univariate models revealed trends (p < 0.20) regarding age in Croatia, Italy, Portugal and Spain, where younger children presented for SA at the hospital during the second period (OR = 0.86 [95% CI: 0.76–0.97], p = 0.017; 0.88 [95% CI: 0.76–1.01], p = 0.068, 0.92 [95% CI: 0.82–1.04], p = 0.19 and 0.92 [95% CI: 0.85–1.01], p = 0.071, respectively), where in France they were older during the second period (OR = 1.15 [95% CI: 1.03–1.28], p = 0.013). (Table 2). In the multivariate analysis children tended to be older during the second period in France (aOR = 1.2 [95% CI:1.06–1.36], p = 5.5e-3), but younger in Italy (aOR = 0.76 [95% CI: 0.63–0.91], p = 4.0e-3). (Table 2). In the multilevel analysis of the whole sample, compared with previously, children after the breakpoints tended to be younger (OR = 0.96 [95% CI: 0.91–1.01], p = 0.064) (Table 3), but the difference is modest, and significance is lost through multivariate model analysis.

#### Sex

The proportion of females increased after SA breakpoints in all studied countries (Table 1). Yet, it only reached statistical significance in two countries, Croatia (OR = 4.61 [95% CI: 2.57; 8.75], p = 8.6e-7 and aOR = 6.32 [95% CI: 2.71; 16.91], p = 6.6e-5) and Spain (OR = 2.19 [95% CI: 1.45; 3.4], p = 3.0e-4 and aOR = 2.13 [95% CI: 1.4; 3.32], p = 5.5e-4) (Table 2). Multilevel analysis confirmed this highly significant (p < 0.01) trend (OR = 1.82 [95% CI: 1.45; 2.29], p = 2.1e-7); aOR = 1.77 [95% CI: 1.34; 2.34], p = 6.0e-5 (Table 3). Although females were overrepresented during the whole studied period, this significantly increased during periods of SA peaks.

#### SA methods

The proportion of self-poisoning was lower in Croatia and France (aOR = 3.57 [95% CI: 1.96–6.66], p = 4.3e-5 and aOR = 1.84 [95% CI: 1.21–2.83], p = 4.9e-3, respectively) during the second period. Multilevel analysis confirmed this change and more SA methods “other than self-poisoning” (OR = 1.34 [95% CI: 1.10–1.63], p = 4.1e-3; aOR = 1.34 [95% CI: 1.05–1.7], p = 0.018) (Table 3) were used during the second period.

#### History of family mental illness and previous ER visit

During the second period, children with SA were less likely to have a family history of mental illness in Portugal and Spain (aOR = 0.32 [95% CI: 0.120.81], p = 0.017 and aOR = 0.73 [95% CI: 0.55–0.96], p = 0.025 respectively) (Table 2). Multilevel analysis showed no significance in family history of mental illness between the two studied periods (Table 3). The proportion of those who had had a previous ER visit for SA did not change significantly across the studied periods, neither in single countries nor at the general level.

#### Impact of contingency measures in Europe and SA in children

**Fig. 2:**
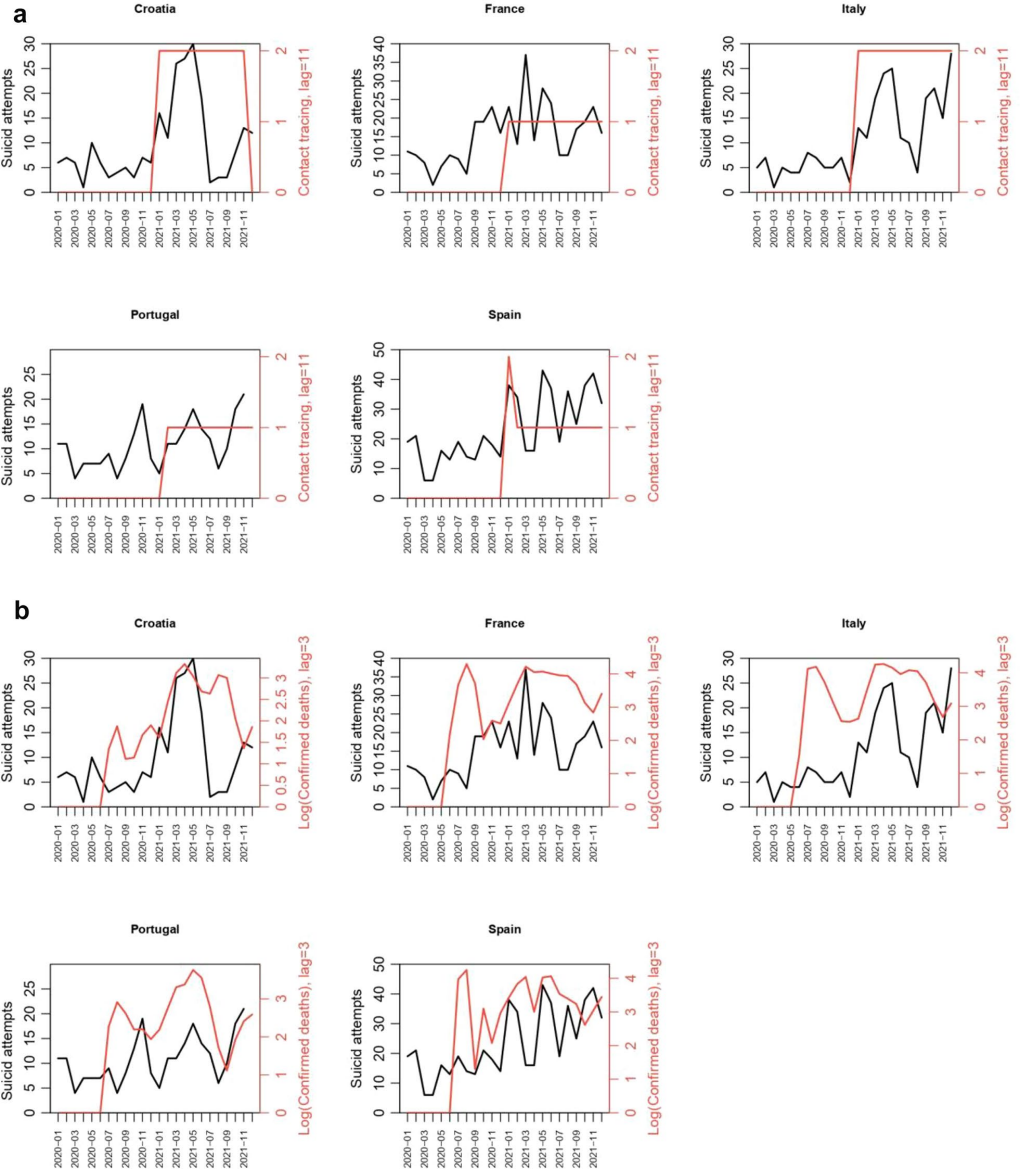
Graphical representation—association between the environmental indicators and the number of SA over time by country. **a** Contact tracing with an 11-month lag (in red), and the number of SA reported (in black). 0 = no contact-tracing, 1 = not done for all cases, 2 = done for all identified cases. The variable was shifted by 11 months (lag used in the statistical analysis) to make the changes coincide with the number of SA observed. **b** Reported number of COVID-19 deaths on a logarithmic scale with a 3-month lag, in red, and the number of SA reported (in black). The variable was shifted by 3 months (lag used in the statistical analysis) to make the changes in value coincide with the number of SA observed

In the univariate analysis, OxCGRT indicators related to contingency measures affected the incidence of SA. We identified ‘optimal lags’ for all indicators, associated with incidence rate ratios (IRR) significantly > 1, with the exception of restrictions of gatherings, international travels, and facial covering (Table S2 Supplementary Material). Among the quantitative variables, the highest IRR among the lagged indicator we explored was the monthly number of COVID-19 deaths (IRR = 1.28 [95% CI: 1.22–1.33], p = 1.7e-7) (Table S2) with a 3-months lag. When considering the qualitative variables, we obtained IRR > 3 for the restrictions on very large gatherings (IRR = 3.12 [95% CI: 1.40-; 4.84], p = 1.2e-3), quarantine arrivals from some or all regions (IRR = 4.6 [95% CI: 2.1; 7.11], p = 7.9e-5), and comprehensive contact tracing done for all identified cases (IRR = 3.05 [95% CI: 2.43–3.66], p = 1.5e-7) (Table S2). Finally, the forward selection process selected two indicators in the multivariate model. The first corresponds to the worsening of the pandemic which is represented in our study by the logarithmic number of COVID-19 deaths, and was associated with an increased number of SA (IRR = 1.13 [1.07; 1.2], p = 7.4e-4) after a 3-months delay. The second, the beginning of the pandemic, which is represented by the implementation of partial and complete contact tracing policies, that were associated with an increased number of SA after an 11-month delay (IRR = 1.37 [1.1; 1.65], p = 8.3e-3; IRR = 2.24 [1.71; 2.77], p = 1.5e-5, respectively) (Table 4; Fig. 2). We also observed that the model was significantly affected by seasonality, data overdispersion and, more importantly, there seem to be additional, country-specific factors that modulate the number of SA (Table 4).

**Table 4:** Parameters estimates with 95% confidence interval affecting significantly the risk of suicide attempts in children based on a negative-binomial regression model

Parameters	Variables	Incidence Rates Ratio [95% confidence interval]	p-value
Seasonality parameters^a^	*sin*(2*πt*/12)	0.034 [-0.05; 0.11]	0.43
	*cos*(2*πt*/12)	0.20 [0.11; 0.29]	5.4e-4
	*sin*(4*πt*/12)	-0.19 [-0.27; -0.10]	6.1e-4
	*cos*(4*πt*/12)	-0.15 [-0.23; -0.07]	3.8e-3
Main governmental measures to follow the COVID-19 pandemic	Contact tracing, lag = 11 months		
	Partial	1.37 [1.1; 1.65]	8.3e-3
	Complete	2.24 [1.71; 2.77]	1.5e-5
	Confirmed deaths, lag = 3 months		
		1.13 [1.07; 1.2]	7.4e-4
Country-specific intercepts^b^	Croatia	4.62 [3.82; 5.42]	2.2e-10
	Italy	4.34 [3.53; 5.15]	9.8e-10
	Portugal	7.57 [6.47; 8.67]	7.5e-13
	France	8.84 [7.52; 10.16]	4.0e-13
	Spain	13.64 [11.84; 15.44]	7.9e-15
Data overdispersion		0.06 [0.03; 0.09]	< 2.2e-16

^a^To adjust for the seasonal variations of the SA time-series, we added 4 sine–cosine dummy variables in the model corresponding to a sine–cosine decomposition (Fourier régression) of this seasonality function: $Seas(t)$=${\delta }_{1}sin \left(\frac{2\pi t}{12}\right)+{ \gamma }_{1}cos \left(\frac{2\pi t}{12}\right){+\delta }_{2}sin \left(\frac{4\pi t}{12}\right)+{ \gamma }_{2}cos \left(\frac{4\pi t}{12}\right)$, with ${\delta }_{1}$, ${\gamma }_{1}$ and ${\delta }_{2}$, ${\gamma }_{2}$. Seasonality parameters estimated by the model for which values are presented in the table. At each time $t$ in the model, the number of SA is multiplied by $exp(Seas(t)$) (see Supplementary Material S1)

^b^Reference value for each country

## Discussion

Overall, our results confirmed a delayed increase in SA during the COVID-19 pandemic in children and adolescents in several European countries, with a quite similar sudden break in the incidence, 6 or 10 months after the beginning of the pandemic. We also reported that the pandemic by itself has modified the clinical profile of children and adolescents who attempted suicide during this period. The burden of the pandemic was not only a modifier of the vulnerability threshold of SA in children but had its own effect on specific vulnerable groups. Finally, our results provided new insight into the relationship between contingency measures in Europe and SA in children. The severity of the pandemic by itself emerged as the strongest predictor in the rise of SA in children and adolescents, with a 3-month lagged effect of the monthly number of COVID-19 deaths (reflecting the severity of the pandemic, and an 11- month lagged effect for the contact tracing indicator (reflecting the pandemic duration onset).

### Trends in SA

Time series of SA in all the countries involved in the study (Croatia, Denmark, France, Italy, Portugal, and Spain) are aligned with previous findings in the world [5–7]. During the COVID-19 pandemic, an initial decline of SA was followed by a significant increase several months later, either in September 2020 (beginning of the school year in Europe) or January 2021 (beginning of a large vaccination campaign in Europe). Although the seasonal variation was globally kept with the expected peak of SA during spring and the nadir at the end of summer [17, 18], we however observed an important exception during the spring of 2020 where SA were exceptionally low. In Europe, this equaled the earlier months of lockdown, and low rates of SA in this time period may be a result of not only increased parental supervision but also difficulties and barriers in accessing urgent mental health care [19], necessarily resulting in a decrease in the number of recorded attempts.”

### Childrens’ profiles before and after the increase in SA

Slightly different patient profiles were observed during the first period and the second (i.e., after the breakpoints) in the five studied countries (all but Denmark) and differences might be difficult to interpret. The results from the multilevel analysis (i.e., summarizing all included countries) showed that patients were more prone to use other SA methods than self-poisoning during SA peaks. Indeed, self-poisoning is amongst the less fatal means, with estimated fatality ratios of 1.5% (while hanging accounts for 61.4%) [20]. Although our sample did not allow for a more in-depth analysis, it is possible that there was a change in fatality ratios before and after the breakpoints. Moreover, if an overrepresentation of girls was seen before the pandemic, it became more striking during the second period, as supported by other studies across the globe [2, 21–24]. The pandemic by itself modified clinical aspects in patients’ profiles of children and adolescents who attempted suicide during this period. Adolescent girls might have been especially vulnerable for developing suicidal ideation during the COVID-19 pandemic. Regarding risk explanatory factors, excessive internet use may contribute to SA in children and adolescents [25, 26] with a bigger influence on girls [27]. And as in similar past pandemics, the risk of exposure to physical and sexual violence increased [19, 28], affecting more girls than boys. As such, girls are a target population for which effective preventive strategies should be implemented [22].

### Impact of contingency measures in Europe and SA in children

In our study, the “contact tracing” indicator was an earlier marker of the potential increase on SA at the midterm (Fig. 2a), and timely matches the breakpoints in the number of SA we reported in several European countries (Fig. 1). The contact-tracing policies were implemented early during the pandemic and maintained throughout its duration (Fig. 2a), serving as a marker of the pandemic’s onset. If our findings are validated, the 11-month delay identified could serve as a sort of countdown from the beginning of the pandemic, allowing us to tailor SA prevention strategies for children and adolescents. Understanding this time lag may highlight a specific period of vulnerability, allowing us to adjust mental health services to meet the anticipated needs.

Consistent with our intuition, the worsening of the pandemic, suggested by the cumulative effect of the number of deaths, led to a shorter lag, measured at 3 months in our study (Fig. 2b). The time between the occurrence of a stressful, negative event and the consequence for mental health is clearly dependent on the potential impact on the individual’s life [29]. In this context, the rise in COVID-19-related deaths, posing an additional threat and likely intensifying the stress burden in vulnerable children, led to a shortening of the estimated time from pandemic onset to peak SA incidence. This observation is notably consistent with the traditionally considered 1-month lag in post-traumatic stress disorder in children, particularly when the stress experienced by the child is deemed significant and vital [29]. Identifying the time between a stressful event and the emergence of clinical symptoms is a major challenge in mental health if we are to develop better primary or secondary prevention strategies. In the case of post-traumatic stress disorder, this improved understanding of temporality has made it possible to target therapeutic strategies and patient follow-up more effectively. In our article, we also attempt to better define the temporality between the emergence of an infectious pandemic—in this case, COVID-19—and the increase in suicide risk in children and adolescents. These two precise timeframes provide a fundamental indication of the urgency of the preventive measures that need to be put in place to prevent suicidal risk in children and adolescents. In particular, the 11-month threshold provides a reassuring long-term trend for health authorities, as it offers the opportunity to implement a medium-term prevention policy that will reach as many people as possible through a variety of information channels. The 3-month threshold is very different, however, and underlines the urgency of the interventions that need to be put in place to curb the risk of suicide attempts among children and adolescents in the short term. It creates a real warning signal for parents and means that care in specialist centers can be better adapted to avoid overloading emergency and hospitalization services.

### Study limitations

Our findings were limited for several reasons. First, the studied European countries adopted similar measures and around the same time period, due to a harmonization of European health policies. This resulted in a relatively homogenous sample, limiting our abilities to contrast the inter-country OxCGRT indicators. This could explain why no single variable, which was examined in the study, was found to have a significant impact on the incidence of SA during the pandemic (ex. stay-at-home requirements policies, school closure), despite being repeatedly identified as major precipitating events on SA in children during the pandemic [9]. Second, from our findings, one could argue that the imposition of lockdown measures alone led to an increase in SA, as indicated by the 11-month lag. However, it is equally plausible that the surge in SA is linked to the severity of the pandemic and, by removing the lockdown measures, SA patterns could have been potentially exacerbated. Further research is recommended as the causal relationship between lockdown measures as a whole and SA cannot be definitively determined by this study alone.

## Conclusions

In summary, our research emphasizes the influence of external factors on pediatric SA during the COVID-19 pandemic. Consequently, if a similar pandemic were to occur, we anticipate a subsequent increase in SA in this population; much like the aftershocks of earthquakes, we can expect a secondary wave. The period between the onset of pandemic and the increase in SA will present an opportunity for prevention, through targeted interventions aimed at especially vulnerable groups (e.g., girls).

### Supplementary Information

Supplementary File1 (Docx 41 Kb)

## Data Availability

The analytical database of this study can be obtained from the corresponding author under reasonable request. OxCGRT indicators are free and available online: https://github.com/OxCGRT/covid-policy-tracker.

## Abbreviations

SA: Suicide attempts
COVID 19: Coronavirus disease 2019
OxCGRT: Oxford COVID-19 government response tracker
PELT: Pruned exact linear time (Method)
BIC: Bayesian information criteria (Method)
