# Supplementary material for: Pediatric suicide attempts lagged during the COVID-19 pandemic: a European multicenter study
Source: Child Adolesc Psychiatry Ment Health. 2024 Aug 7;18:98. doi: 10.1186/s13034-024-00784-2 (PMC11308394; doi:10.1186/s13034-024-00784-2)
Supplement: Supplementary file 1 — Supplementary File1 (Docx 41 Kb) [file 13034_2024_784_MOESM1_ESM.docx]

# Supplementary material

**Table S1: participating centers in the study.**

| **Country** | **Department** | **Age Cut-off** | **Type of data availability** |
| --- | --- | --- | --- |
| Croatia | Psychiatric Hospital for Children and Youth, Zagreb | 18 | Paper and digital database |
| Denmark | National data | 18 | Digital database |
| France | Robert Debré University Hospital, Paris. | 18 | Paper and digital database |
| Italy | Bambino Gesù Children’s University Hospital, Rome | 18 | Paper and digital database |
| Portugal | D. Estefânia University Hospital, Lisbon | 18 | Digital database |
| Spain | Saint Joan de Déu University Hospital, Barcelona. | 18 | Digital database |

## S1: Association between the number of SA and OxCGRT indicators - Methods

### Indicators included

We included all the OxCGRT indicators publicly available on <https://github.com/OxCGRT/covid-policy-tracker>. As the indicators were given daily, we aggregate them to obtain monthly indicators to match the SA da, as follow:

- For data related to the evolution of the pandemic (log_10_(Confirmed cases), log_10_(Confirmed deaths)), we made a monthly aggregation by summing the daily observations over the month.
- For other quantitative indicators (Stringency index, Containment health index, Government response index, Economic support index), we used monthly means.
- For qualitative indicators (all other indicators), we used the value that was present majority during the month.

For the purpose of the analysis, we added two additional variables to the set of OxCGRT indicators:

- A trend, to catch a possible increase in SA not related to OxCGRT indicators during the period of our analysis.
- The elapsed time (in months) since the beginning of containment measures related to the pandemic in Europe, i.e. February 2020, to account for the potential deleterious impact of the sustainability of the pandemic over time.
- A pandemic period indicator variable equal to 1 if the current month is after February 2020, 0 otherwise.

The list of indicators we used are available in Table S2and a detailed list of OxCGRT indicators with description can be found at <https://github.com/OxCGRT/covid-policy-tracker/blob/master/documentation/codebook.md>

### Model

We used a negative-binomial model to explore the links between the number of SA observed at time $t$ and OxCGRT indicators. The basic model can be described by the following equations:

$$Y_{ct}\sim NegBin\left( \mu_{ct}, \psi\right)$$

$loglog \left( \mu_{ct} \right) =\alpha_{c}+\sum_{i} \beta_{i}X_{i,c,t-l}+Seas(st)$

With $Y_{ct}$ the number of SA reported in country $c$ at time $t$, $\mu_{ct}$ the expected mean, and $\psi$ the overdispersion parameter. $\alpha_{i}$ are country-specific intercepts, $X_{i,c,t}$ the value observed for the OxCGRT indicator $i$ in country $c$ at time $t$, and $\beta_{i}$ the incidence rate ratios associated with this indicator. As we expected a lag between the changes in the OxCGRT indicators and the effect on the number of reported SA, we made $loglog \left( \mu_{ct} \right)$ depends on $X_{i,c,t-l}$, with $l$ being the lag.

We also included a variable to account for the annual seasonality we expect this kind of time series, using a sinus/cosinus decomposition under the form: $Seas(ts)=\sum_{s=1}^{2} \delta_{s}sinsin \left( \frac{2s\pi t}{12} \right) +\gamma_{s}coscos \left( \frac{2s\pi t}{12} \right)$. We limited the number of parameters in the seasonality to 4 using $s=2$ to limit the number of nuisance parameters.

### Indicator selection

We performed a variable selection in two steps. We used the Bayesian Information Criteria (BIC) (1) instead of Akaike Information Criteria (AIC)(2) as it tends to select more parsimonious models, and we have 10 nuisance parameters to estimate (5 intercepts, 4 parameters for the seasonality, and 1 for overdispersion).

Step 1: we selected the optimal lag for each indicator $i$. To do this, we fitted for the latter the following univariate model for $l\in\left\{ 0,.. . ,N \right\}$:

$$Y_{ct}\sim NegBin\left( \mu_{ct}, \psi\right)$$

$loglog \left( \mu_{ct} \right) =\alpha_{c}+\beta_{i}X_{i,c,t-l}+S$

Then, we determined the optimal lag $l$ as the one minimizing the BIC. We limited the maximum lag to $N$=18 months.

Step 2: using for each variable the optimal lag determined at step 1, we performed a forward selection, i.e. starting from the empty model (including only the nuisance parameters):

1. We sought which indicator was associated with the lowest BIC when added in the model and we added it to the model.
2. We performed i. again until no indicator was able to decrease the BIC.

The last model (i.e. with the lowest BIC) was used as the final multivariate model.

### Software

Analysis were implemented on R using the “surveillance” package (3).

## S2: Optimal lags found for the association between the number of SA and the indicators, and results of the univariate analysis

Table S2: Optimal lags for each indicator.

| **Variable** | **Optimal lag (in months)** | **IRR [95% CI]**  **Univariate analysis**† | **p-value** |
| --- | --- | --- | --- |
| Stringency index | 11 | 1.01 [1.01 ; 1.01] | 2.4e-7 |
| Containment health index | 10 | 1.01 [1.01 ; 1.01] | 1.2e-7 |
| Government response index | 10 | 1.01 [1.01 ; 1.01] | 9.6e-8 |
| Economic support index | 9 | 1.01 [1.01 ; 1.01] | 1.0e-7 |
| onfirmed COVID cases, logarithmic scale | 4 | 1.18 [1.15 ; 1.21] | 1.5e-7 |
| Confirmed COVID deaths, logarithmic scale | 3 | 1.28 [1.22 ; 1.33] | 1.7e-7 |
| School closing | 10 |  |  |
| *Recommend closing or all schools open with alterations resulting in significant differences compared to non-Covid-19 operations* |  | 1.72 [1.36 ; 2.08] | 2.1e-4 |
| *Require closing (only some levels or categories, eg just high school, or just public schools)* |  | 2.51 [2.07 ; 2.95] | 1.4e-7 |
| *Require closing all levels* |  | 2.34 [1.84 ; 2.84] | 2.8e-6 |
| Workplace closing | 10 |  |  |
| *Recommend closing (or recommend work from home) or all businesses open with alterations resulting in significant differences compared to non-Covid-19 operation* |  | 2.4 [1.45 ; 3.35] | 8.3e-4 |
| *Require closing (or work from home) for some sectors or categories of workers* |  | 2.3 [1.96 ; 2.64] | 5.2e-8 |
| *Require closing (or work from home) for all-but-essential workplaces (eg grocery stores, doctors)* |  | 1.94 [1.42 ; 2.46] | 3.0e-4 |
| Cancel public events | 12 |  |  |
| *Recommend cancelling* |  | 1.68 [1.15 ; 2.21] | 7.7e-3 |
| *Require cancelling* |  | 2.41 [2.03 ; 2.79] | 1.4e-7 |
| Restrictions on gatherings | 4 |  |  |
| *Restrictions on very large gatherings (the limit is above 1000 people)* |  | 3.12 [1.4 ; 4.84] | 1.2e-3 |
| *Restrictions on gatherings between 101-1000 people* |  | 1.35 [0.85 ; 1.85] | 0.14 |
| *Restrictions on gatherings between 11-100 people* |  | 2.11 [1.42 ; 2.8] | 5.1e-4 |
| *Restrictions on gatherings of 10 people or less* |  | 2.23 [1.91 ; 2.56] | 3.2e-8 |
| Close public transport |  |  |  |
| *Recommend closing (or significantly reduce volume/route/means of transport available)* | 10 | 2.07 [1.63 ; 2.5] | 3.1e-5 |
| Stay at home requirements | 10 |  |  |
| *Recommend not leaving house* |  | 2.68 [2.03 ; 3.34] | 4.1e-6 |
| *Require not leaving house with exceptions for daily exercise, grocery shopping, and 'essential' trips* |  | 2.2 [1.82 ; 2.58] | 1.2e-6 |
| Movement restrictions | 10 |  |  |
| *Recommend not to travel between regions/cities* |  | 2.2 [1.59 ; 2.81] | 1.3e-4 |
| *Internal movement restrictions in place* |  | 2.47 [2 ; 2.95] | 8.8e-7 |
| International travel | 10 |  |  |
| *Screening arrivals* |  | 1.45 [0.92 ; 1.98] | 0.066 |
| *Quarantine arrivals from some or all regions* |  | 4.6 [2.1 ; 7.11] | 7.9e-5 |
| *Ban arrivals from some regions* |  | 2.15 [1.85 ; 2.45] | 4.0e-8 |
| *Ban on all regions or total border closure* |  | 2.17 [1.44 ; 2.91] | 4.9e-4 |
| Income support | 9 |  |  |
| *Government is replacing less than 50% of lost salary (or if a flat sum, it is less than 50% median salary)* |  | 1.96 [1.58 ; 2.33] | 1.6e-5 |
| *Government is replacing 50% or more of lost salary (or if a flat sum, it is greater than 50% median salary)* |  | 2.37 [1.96 ; 2.77] | 3.8e-7 |
| Public information campaigns - coordinated public information campaign (eg across traditional and social media) | 11 | 2.24 [1.94 ; 2.54] | 1.3e-7 |
| Testing policy | 11 |  |  |
| *Only those who both (a) have symptoms AND (b) meet specific criteria (eg key workers, admitted to hospital, came into contact with a known case, returned from overseas)* |  | 2.06 [1.49 ; 2.63] | 2.0e-4 |
| *Testing of anyone showing Covid-19 symptoms* |  | 2.38 [1.97 ; 2.78] | 1.7e-7 |
| *Open public testing (eg "drive through" testing available to asymptomatic people)* |  | 2.06 [1.64 ; 2.48] | 1.0e-5 |
| Contact tracing | 11 |  |  |
| *Limited contact tracing; not done for all cases* |  | 1.88 [1.59 ; 2.16] | 3.2e-6 |
| *Comprehensive contact tracing; done for all identified cases* |  | 3.05 [2.43 ; 3.66] | 1.5e-7 |
| Facial coverings | 7 |  |  |
| *Recommended* |  | 2.43 [1.3 ; 3.57] | 2.1e-3 |
| *Required in some specified shared/public spaces outside the home with other people present, or some situations when social distancing not possible* |  | 1.33 [0.95 ; 1.71] | 0.068 |
| *Required in all shared/public spaces outside the home with other people present or all situations when social distancing not possible* |  | 2 [1.6 ; 2.41] | 1.0e-5 |
| *Required outside the home at all times regardless of location or presence of other people* |  | 2.53 [2.09 ; 2.97] | 5.2e-8 |
| Vaccination policy | 2 |  |  |
| *Availability for TWO of following: key workers/ clinically vulnerable groups (non elderly) / elderly groups* |  | 2.95 [2.21 ; 3.68] | 6.5e-7 |
| *Availability for ALL of following: key workers/ clinically vulnerable groups (non elderly) / elderly groups* |  | 2.25 [1.52 ; 2.98] | 2.3e-4 |
| *Availability for all three plus partial additional availability (select broad groups/ages)* |  | 1.59 [1.11 ; 2.07] | 9.1e-3 |
| *Universal availability* |  | 2 [1.54 ; 2.46] | 3.7e-5 |
| Protection of elderly people | 10 |  |  |
| *Recommended isolation, hygiene, and visitor restriction measures in LTCFs and/or elderly people to stay at home* |  | 2.04 [1.52 ; 2.57] | 1.1e-4 |
| *Narrow restrictions for isolation, hygiene in LTCFs, some limitations on external visitors and/or restrictions protecting elderly people at home* |  | 2.28 [1.84 ; 2.71] | 1.2e-6 |
| *Extensive restrictions for isolation and hygiene in LTCFs, all non-essential external visitors prohibited, and/or all elderly people required to stay at home and not leave the home with minimal exceptions, and receive no external visitors* |  | 2.15 [1.66 ; 2.64] | 1.7e-5 |
| Trend | 12 | 1.04 [1.03 ; 1.04] | 7.2e-7 |
| Pandemic period | 10 | 2.21 [1.92 ; 2.5] | 1.2e-7 |
| Pandemic duration | 2 | 1.06 [1.05 ; 1.07] | 2.2e-7 |

IRR=Incidence Rate Ratio. †Univariate models included adjustment for seasonality using a sinus/cosinus decomposition as explained in Supplementary Material S1. Briefly, for most indicators related to containment measures or economic support, we found an optimal association with the number of SA for lags ranging from 9 to 11 months. This is consistent with breakpoints found previously (around January 2021 in most countries), as most measures were implemented in European countries from February/March 2020.

**References**

1. Schwarz G. Estimating the dimension of a model. Ann Stat. 1978;461–4.

2. Akaike H. Information theory and an extension of the maximum likelihood principle. In: 2nd International Symposium on Information Theory. Akadémiai Kiadó Location Budapest, Hungary; 1973. p. 267–81.

3. Meyer S, Held L, Höhle M. Spatio-Temporal Analysis of Epidemic Phenomena Using the R Package surveillance. J Stat Softw. 2017;77(11).
